# Astragaloside IV Improves High-Fat Diet–Induced Hepatic Steatosis in Nonalcoholic Fatty Liver Disease Rats by Regulating Inflammatory Factors Level via TLR4/NF-κB Signaling Pathway

**DOI:** 10.3389/fphar.2020.605064

**Published:** 2021-01-29

**Authors:** Ying-Li Liu, Qiu-Zan Zhang, Yan-Rong Wang, Li-Na Fu, Jing-Shu Han, Jing Zhang, Bang-Mao Wang

**Affiliations:** ^1^ Gastroenterology, the Fourth Central Clinical College, Tianjin Medical University, Tianjin, China; ^2^ Gastroenterology, Tianjin Fourth Central Hospital, Tianjin, China; ^3^ Gastroenterology, Tianjin Medical University General Hospital, Tianjin, China

**Keywords:** astragaloside IV, non-alcoholic fatty liver disease, toll like receptor 4, nuclear factor-kappa B, myeloid differentiation factor 88

## Abstract

**Objective**: Astragaloside IV (AS-IV) is the primary bioactive component purified from *Astragalus membranaceus* which is one of the traditional Chinese medicines. Research studies found that AS-IV has significant pharmacological effects on focal cerebral ischemia/reperfusion, cardiovascular disease, pulmonary disease, liver cirrhosis, and diabetic nephropathy, but little is known about the effects of AS-IV on nonalcoholic fatty liver disease (NAFLD). In this study, we investigated whether AS-IV has beneficial effects on NAFLD in rats and its potential mechanisms.

**Methods**: Male SD rats were fed with high-fat diet (HFD) for 12 weeks to establish NAFLD rat model, and then, the rats were divided into five groups. The control group rats were fed with normal diet for 12 weeks and then were given normal saline (1.0 ml kg^−1^ day^−1^) by intragastric administration for 4 weeks. The model group rats were fed with HFD for 12 weeks and then were given normal saline (1.0 ml kg^−1^ day^−1^) by intragastric administration for 4 weeks. The AS-IV-L, AS-IV-M, and AS-IV-H groups were treated with 20, 40, and 80 mg kg^−1^ day^−1^ of AS-IV by intragastric administration for 4 weeks and given HFD diet. Then, we detected serum transaminase (ALT, AST), blood lipid (TG, TC), inflammatory cytokines (IL-6, IL-8 and TNF-α), liver histology(NAFLD activity score), TLR4/MyD88 signaling pathway in liver tissue.

**Results**: We found AS-IV significantly reduced serum levels of AST, ALT, TG, TNF-α, IL-6, and IL-8 in NAFLD rats and downregulate the expression of TLR4 mRNA, MyD88 mRNA, NF-κB mRNA, and proteins in liver tissue. Moreover, AS-IV could significantly reduce the NAFLD activity score of NAFLD rat liver.

**Conclusion**: In this study, we demonstrated that AS-IV have a protective effect on NAFLD by inhibiting TNF-α, IL-6 and IL-8 levels and down-regulating TLR4, MyD88 and NF-κB expression in rat liver tissues.

## Introduction

Nonalcoholic fatty liver disease (NAFLD) is the most common chronic liver disease worldwide. NAFLD includes three subtypes: nonalcoholic fatty liver (NAFL), nonalcoholic steatohepatitis (NASH), and related liver cirrhosis. NAFL can develop into NASH, while NASH can gradually develop into liver cirrhosis and liver cancer (Diehl and Day, 2017). The prevalence of NAFLD in ordinary adults is between 6.3% and 45%, and the average prevalence is as high as 25.24% (Younossi et al., 2016). An epidemiological survey from Shanghai, Beijing, and other regions in China showed that the prevalence of NAFLD in ordinary adults diagnosed by B-type ultrasonography had increased from 15% to more than 31% over a 10-year period (Zhu et al., 2015). The pathogenesis of NAFLD has not been elucidated completely, and the theory of "multiple strikes" is widely accepted by professionals at present (Tilg and Moschen, 2010). Recently, the mechanism of innate immunity in NAFLD pathogenesis has received more and more attention. Studies had indicated that TLR4 signaling pathway was one of the key factors in the pathogenesis of different chronic liver diseases including NAFLD (Soares et al., 2010; Roh and Seki, 2013) and was associated with the progression of NASH (Zhang et al., 2013; Kapil et al., 2016). Singh et al. (2011) revealed that the expression of TLR4 mRNA and its protein in normal liver tissues was lower than that in NASH patients. This suggested the importance of immunological inhibition and immune tolerance in the normal liver. Sharifnia et al. (2015) found that the expression of TLR4 mRNA and interferon regulatory factor-3 (IRF-3) mRNA in the liver of NASH patients were significantly increased compared with NAFLD patients. Furthermore, the expression of TLR4 and its downstream mediators were upregulated after treated with palmitate and lipopolysaccharide (LPS). It indicated that TLR4 had vital function on pathogenesis of NASH and was one of the important factors related to LPS sensitivity and fatty acid damage.

Present studies have found that innate immunity plays an important role in NAFLD pathogenesis. TLRs are a series of pattern recognition receptors and play the crucial role in the activation of the innate immune system by identifying pathogen-associated molecular patterns (PAMPs) (Leifer and Medvedev, 2016; Vidya et al., 2018). TLR4 is an important member of TLRs family. It is an endotoxin recognition receptor that mediates innate immunity. It is a bridge between innate immunity and acquired immunity of the human body (Takeda and Akira, 2015). TLR4 is mainly located on the surface of the cell membrane and is the receptor of intestinal-derived endotoxin lipopolysaccharide (LPS) of Gram-negative bacteria (Akira et al., 2006). TLR4 can initiate a series of injury-related immune responses (Erridge, 2010; Wakefield et al., 2010). TLR4 interacts with its downstream adaptor molecule myeloid differentiation factor 88 (MyD88) and then activates nuclear factor-κB (NF-κB) transcription factors to produce and release cytokines. As one of the important pathways associated with inflammatory response, TLR4/NF-κB signal transduction pathway activation can lead to a large number of expressions of inflammatory factors including TNF-α, IL-1, IL-6, IL-8, and adhesion molecules and then induce a series of inflammatory responses (Snyder and Sundberg, 2014; Mitchell et al., 2016).

**FIGURE 1:**
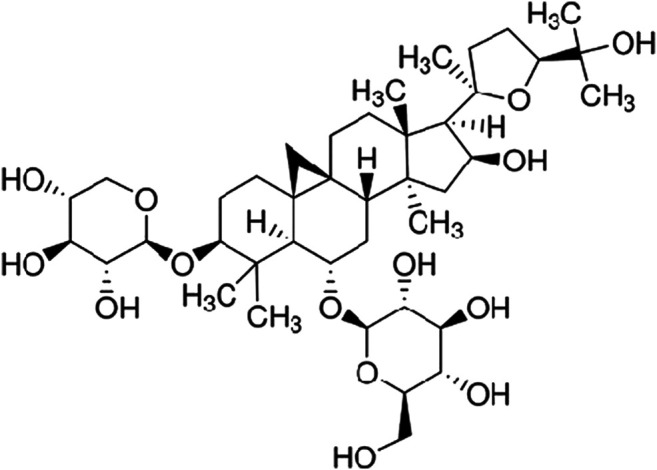
Chemical structure of astragaloside IV.

Until now, there is no common acknowledged therapeutic method to NAFLD, although NAFLD is very common (Diehl and Day, 2017). AS-IV is a monomer component purified from Chinese traditional medicine *Astragalus membranaceus*. The molecular structure was extracted from *Astragalus membranaceus* in 1983 by Japanese scholars Kitagawa et al. (1983). The molecular formula of AS-IV is C14H68O14, and its molecular mass is 784.97 (Figure 1). The bioavailability of AS-IV after p.o. administration is only 3.66% in rats (Zhang et al., 2007), and the low absorption is mainly due to its poor intestinal permeability, high molecular weight, low lipophilicity, and its paracellular transport in Caco-2 cells (Huang et al., 2006). It has been revealed that AS-IV had multiple biological activities, such as anti-inflammatory, antioxidation, lipid-regulating, hypoglycemic, and immunomodulating activities (Ren et al., 2013; Li et al., 2017). It has been shown that AS-IV could play anti-inflammatory role through multiple pathways. AS-IV can regulate cytokines, inflammatory factors, signaling pathways, and apoptosis-related genes which are associated with anti-inflammatory injury (Li et al., 2017). Zhang and Frei (2015) reported that AS-IV could effectively inhibit LPS-induced acute inflammatory responses in different organs of rats by regulating TLR4/NF-κB, reducing TNF-α and IL-6 expression. Zhou et al. (2017b) demonstrated that AS-IV inhibited TLR4/NF-kB signaling pathways in intervening unilateral ureteral obstruction model mice and LPS-induced epithelial cells. Lv et al. (2010) reported that AS-IV could reduce glycogen phosphatase and glucose-6-phosphate levels in the liver, reduce blood glucose and triglyceride levels, and improve insulin resistance in type 2 diabetic mice. However, the impacts of AS-IV on NAFLD have been rarely reported. In this study, our aim is to investigate whether AS-IV can improve HFD-induced hepatic steatosis by inhibiting the expression of TLR4, MyD88, and NF-κB in the liver tissue of NAFLD rats.

## Materials and Methods

### Animals and Treatments

A total of 53 male Sprague Dawley rats (age, 6 weeks; weight, 200 ± 20 g) were purchased from Beijing Vital River Laboratory Animal Technology Co. Ltd. (Beijing, China, NO.SCXK 2016-0011) and raised in the Experimental Animal Center of the Institute of Radiation Medicine, Chinese Academy of Medical Sciences (Tianjin, China). The rats were maintained under standard conditions of temperature (22 ± 2°C) and humidity (50 ± 5%) in a 12 h light/dark cycle. Animals were allowed free access to food and water throughout acclimatization and experimental periods. After 1 week of acclimatization, they were randomly divided into control group (CON, n = 10), model group (MOD, n = 13), and intervention group (n = 30). The control group was fed with normal diet for 12 weeks, followed by 4 weeks of intragastric administration of saline (1.0 ml kg^−1^ day^−1^) and continuous normal diet feeding. The model group and the intervention group were fed high-fat diet (HFD, 88% of normal diet, plus 2% of cholesterol and 10% of lard) for 12 weeks for NAFLD modeling. After 12 weeks, three rats of the model group were randomly selected to confirm the successful modeling of NAFLD by liver histopathological examination and the other 10 rats were subsequently intragastrically administered with 1.0 ml kg^−1^ day^−1^ saline for 4 weeks alongside HFD feeding. The intervention group was then randomly divided into astragaloside IV (Dalian, China, Cat. No. MB 1955) low-dose group (AS-IV-L, n = 10), middle-dose group (AS-IV-M, *n* = 10), and high-dose group (AS-IV-H, *n* = 10). Rats in the intervention groups were intragastrically administered with different concentrations of AS-IV (20 mg kg^−1^ day^−1^ for AS-IV-L, 40 mg kg^−1^ day^−1^ for AS-IV-M, or 80 mg kg^−1^ day^−1^ for AS-IV-H) for 4 weeks, respectively. The weight and food intake of rats in each group were measured weekly. All animal procedures conducted in this study were approved by experimental animal ethics committee, Institute of Radiology, Chinese Academy of Medical Sciences(Ethics number IRM-201905-072).

### Serum Biochemical Analysis

After 16 weeks, all rats were fasted for 12 h with free water. Then, the rats were anesthetized via an intraperitoneal injection of 0.3% sodium pentobarbital (40 mg kg^−1^ day^−1^; Sigma-Aldrich Merck KgaA, Darmstadt, Germany). Samples of blood were collected from the femoral artery before the animals were sacrificed. The serum contents of alanine aminotransferase (ALT), aspartate aminotransferase (AST), triglyceride (TG), and total cholesterol (TC) were tested using an automatic biochemical analyzer (Beckman Coulter, Inc., Brea, CA, USA). Serum levels of TNF-α, IL-6, and IL-8 were detected using ELISA kits of TNF-α (RayBiotech, Atlanta, USA), IL-6 (RayBiotech, Atlanta, USA), and IL-8 (CusabioBiotec, Wuhan, China, Cat. No. CSB-E07273r), following the manufacturer's instructions, respectively. Absorbance at 450 nm was measured using a microplate spectrophotometer (ThermoScientific Multiskan GO).

### Histopathology Analysis

After 16 weeks, all rat livers were collected, part of the liver was fixed in 4% neutral formalin at 4°C overnight, and the liver tissues were prepared into 5 µm liver pathological sections on a microtome. Sections were stained with hematoxylin and eosin (H&E) according to standard techniques. The sections were observed by two blinded experienced pathologists. Twenty high-magnitude visual fields were observed randomly in every section, and the NAFLD activity score (NAS) were obtained under the microscope. NAS was shown in Table 1 (Chalasani et al., 2012).

**TABLE 1:** Nonalcoholic fatty liver disease activity score (NAS).

Score	0	1	2	3
Steatosis	<5%	5–33%	34–66%	>67%
Lobular inflammation	None	<2 foci per 200×	2–4 foci per 200×	>4 foci per 200×
Ballooning change	None	Few	Many	

### Quantitative Real-Time (RT) PCR

Total RNA was extracted from rat liver tissues using TRIzol reagent kit (Applied Biosystems Inc., Carlsbad, USA, Cat. No. 15596–026). cDNA was synthesized from 1 mg of total RNA with a reverse transcriptase kit (Vazyme-biotec, Nanjing, China, Cat. No. R101-01/02). The primer sequences (TSINGKE Biological Technology, Beijing, China) used in the real-time PCR (RT-PCR) assay were shown in Table 2. GAPDH served as an internal reference. SYBR Green qPCR Master Mix (Vazyme-biotech, Nanjing, China, Cat. No. Q111-02) was used for RT-PCR amplification. The cycle conditions were denaturation at 95°C for 10 min followed by 40 repeated annealings at 95°C for 30 s and extension at 60°C for 30 s. The mRNA expression levels were assessed using the 2^−ΔΔCq^ method.

**TABLE 2:** Primer sequences for real-time quantitative PCR.

Name	Primer	Sequence	Size
TLR4	Forward	5′-TAT​CGG​TGG​TCA​GTG​TGC​TT -3′	167 bp
	Reverse	5′-CTC​GTT​TCT​CAC​CCA​GTC​CT -3′	
Myd88	Forward	5′-TTC​GAC​GCC​TTC​ATC​TGC​TA -3′	177 bp
	Reverse	5′-CAT​GCG​ACG​ACA​CCT​TTT​CT -3′	
NF-κBp65	Forward	5′-ACG​CAA​AAG​GAC​CTA​CGA​GA -3′	171 bp
	Reverse	5′-ATG​GTG​CTG​AGG​GAT​GTT​GA -3′	
GAPDH	Forward	5′-ACA​GCA​ACA​GGG​TGG​TGG​AC-3′	253 bp
	Reverse	5′-TTT​GAG​GGT​GCA​GCG​AAC​TT-3′	

### Western Blotting

Detection of liver TLR4, MyD88, and NF-κB p65 protein expression was performed according to the kit (Beyotime Biotechnology, Shanghai, China) manufacturer's instructions. 100mg liver tissue was lyzed in PMSF buffer for 30 min and then centrifuged for 5 min at 12,000 rpm and 4°C. The supernatant of liver tissue was used to quantitatively detect the protein levels. Protein samples (50 μg) from rat liver tissues were separated by 12% SDS-PAGE and electrotransferred to a nitrocellulose membrane, followed by 5% dried skimmed milk blocking at room temperature for 2 h and hybridization overnight at 4°C with the primary antibodies (MYD88 Rabbit Polyclonal antibody, WUHAN SANYING, Wuhan, Hubei, China, Cat. No. 23230-1-AP; NF-κB p65 Rabbit Polyclonal antibody, WUHAN SANYING, Wuhan, Hubei, China, Cat. No. 10745-1-AP; TLR4 Rabbit Polyclonal antibody, WUHAN SANYING, Wuhan, Hubei, China, Cat. No. 19811-1-AP). The membrane was then immunoblotted with secondary antibodies (HRP Conjugated AffiniPure Goat Anti-rabbit IgG, Boster Biological Technology, Wuhan, Hubei, China, Cat. No. BA1054) at 1:50,000 dilution for 2 h at 37°C.The loading control used GAPDH antibody (GAPDH rabbit polyclonal antibody, Goodhere Biological Technology, Zhejiang, China,Cat.No.AB-P-R001). The bands were visualized by the enhanced chemoluminescence (ECL) system. BandScan 5.0 software was used to digitally analyze the strip Gy values for semiquantitative detection.

### Statistical Analysis

SPSS 20.0 statistical software (SPSS, Inc., Chicago, IL, USA) was used for all statistical analyses. Measurement data were expressed as the mean ± standard error of the mean. Data comparisons between two groups used Student's *t*-tests and data comparison among multiple groups used one-way ANOVA analysis. A value of *p* < 0.05 was considered significant difference.

## Results

### The Weight of Rats and Daily Food Intake

**FIGURE 2:**
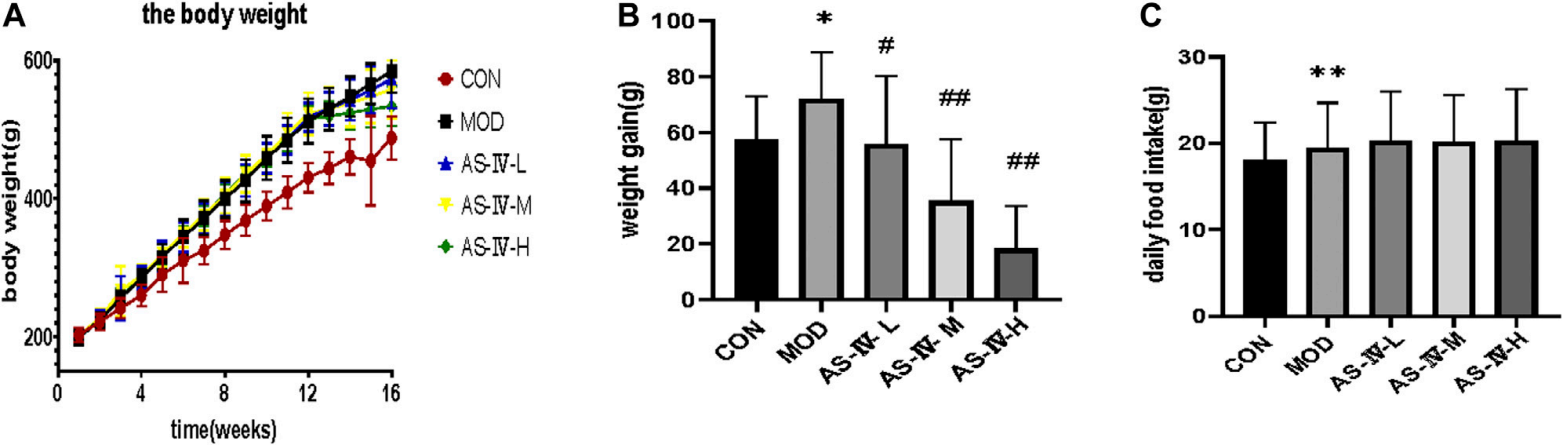
Body weight and daily food intake of rats. **(A)** The body weight of rats. **(B)** The body weight gain after 12 weeks. **(C)** The average daily food intake of rats in different groups. Data present the mean ± SD. Comparisons between two groups use *t*-tests, and data comparison among multiple groups use one-way ANOVA. *p < 0.05 and **p < 0.01 compared with the control group; ^#^ *p* < 0.05 and ^##^ *p* < 0.01 compared with the model group. CON, control group; MOD, model group; AS-IV-L, astragaloside IV low-dose group; AS-IV-M, astragaloside IV middle-dose group; AS-IV-H, astragaloside IV high-dose group.

After 12 weeks of feeding, the body weight of rats fed with normal diet and high-fat diet all increased steadily, while the weight of rats fed with high-fat diet increased significantly faster than that of rats fed with normal diet. After 16 weeks, the weight gain rate of AS-IV-L, AS-IV-M, and AS-IV-H groups were significantly lower than the model group. However, there was no significant difference in the daily food intake among groups. This indicated that AS-IV reduces the weight gain of NAFLD rats, not by reducing their food intake (Figure 2).

### Astragaloside IV Reduces the Serum TG, ATL, and AST Levels of NAFLD Rats

To investigate the effect of astragaloside IV on NAFLD rats, we tested serum TG, TC, ATL, and AST levels of rats of each group at the end of 16 weeks. NAFLD rats showed significantly higher levels of serum TC and TG than the control group. This was consistent with the characteristics of NAFLD. After treated with high-dose AS-IV for 4 weeks, the serum TG level was significantly deceased in NAFLD rats (*p* < 0.01), but the low-dose and middle-dose AS-IV have no obvious effect on reducing the serum TG level. Meanwhile, all the low, middle, and high doses of AS-IV did not reduce the serum TC level. This shows that AS-IV has no obvious effect on the regulation of lipid metabolism.

**FIGURE 3:**
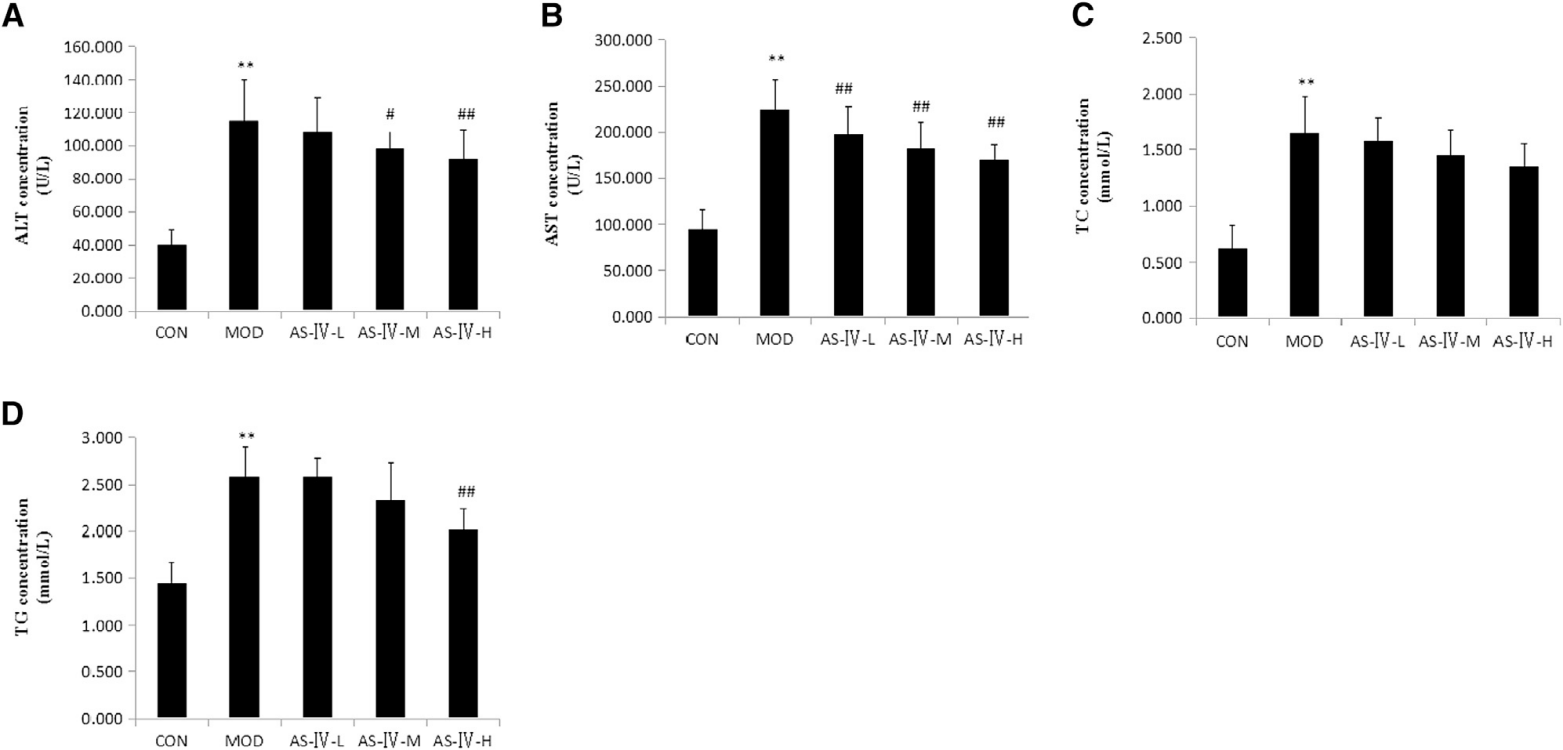
Effects of astragaloside IV on biochemical parameters in HFD-fed rats. CON: 10 SD rats fed with normal diet for 12 weeks and then treated with intragastric administration of saline (1.0 ml/kg/day) and normal diet for 4 weeks. MOD: 10 SD rats fed with HFD for 12 weeks and then treated with intragastric administration of saline (1.0 ml/kg/day) and HFD diet for 4 weeks. AS-IV-L: 10 SD rats fed with HFD for 12 weeks and then treated with intragastric administration of 20 mg kg^−1^ day^−1^ and HFD for 4 weeks. AS-IV-M: 10 SD rats fed with HFD for 12 weeks and then treated with intragastric administration of 40 mg kg^−1^ day^−1^ and HFD for 4 weeks. AS-IV-H: 10 SD rats fed with HFD for 12 weeks and then treated with intragastric administration of 80 mg kg^−1^ day^−1^ and HFD for 4 weeks. **(A)** Serum ALT concentration of each group. **(B)** Serum AST concentration of each group. **(C)** Serum TC concentration of each group. **(D)** Serum TG concentration of each group. Data present the mean±SD. Comparisons between two groups use *t-*tests and data comparison among multiple groups use one-way ANOVA. *p < 0.05 and **p < 0.01 compared with the control group; ^#^ *p* < 0.05 and ^##^ *p* < 0.01 compared with the model group. HFD, high-fat diet; ALT, alanine aminotransferase; AST, aspartate aminotransferase; TC, total cholesterol; TG, triglyceride; CON, control group; MOD, model group; AS-IV-L, astragaloside IV low-dose group; AS-IV-M, astragaloside IV middle-dose group; AS-IV-H, astragaloside IV high-dose group.

The serum AST and ALT levels in the NAFLD rats were significantly higher than those in the control group rats. This was consistent with the characteristics of NAFLD. All of the high-dose (80 mg kg^−1^ day^−1^), middle-dose (40 mg kg^−1^ day^−1^), and low-dose (20 mg kg^−1^ day^−1^) AS-IV treatments showed a significantly decreased level of serum AST in NAFLD rats (*p* < 0.05). Meanwhile, the middle- and high-dose treatments of AS-IV showed a significant decrease in the ALT levels of NAFLD rats (*p* < 0.05). The effect of reduced AST and ALT seemed to be dose-dependent. This suggested that AS-IV can significantly reduce the release of transaminase due to hepatocyte injury (Figure 3).

### Astragaloside IV Improves Hepatic Steatosis in NAFLD Rats

**FIGURE 4:**
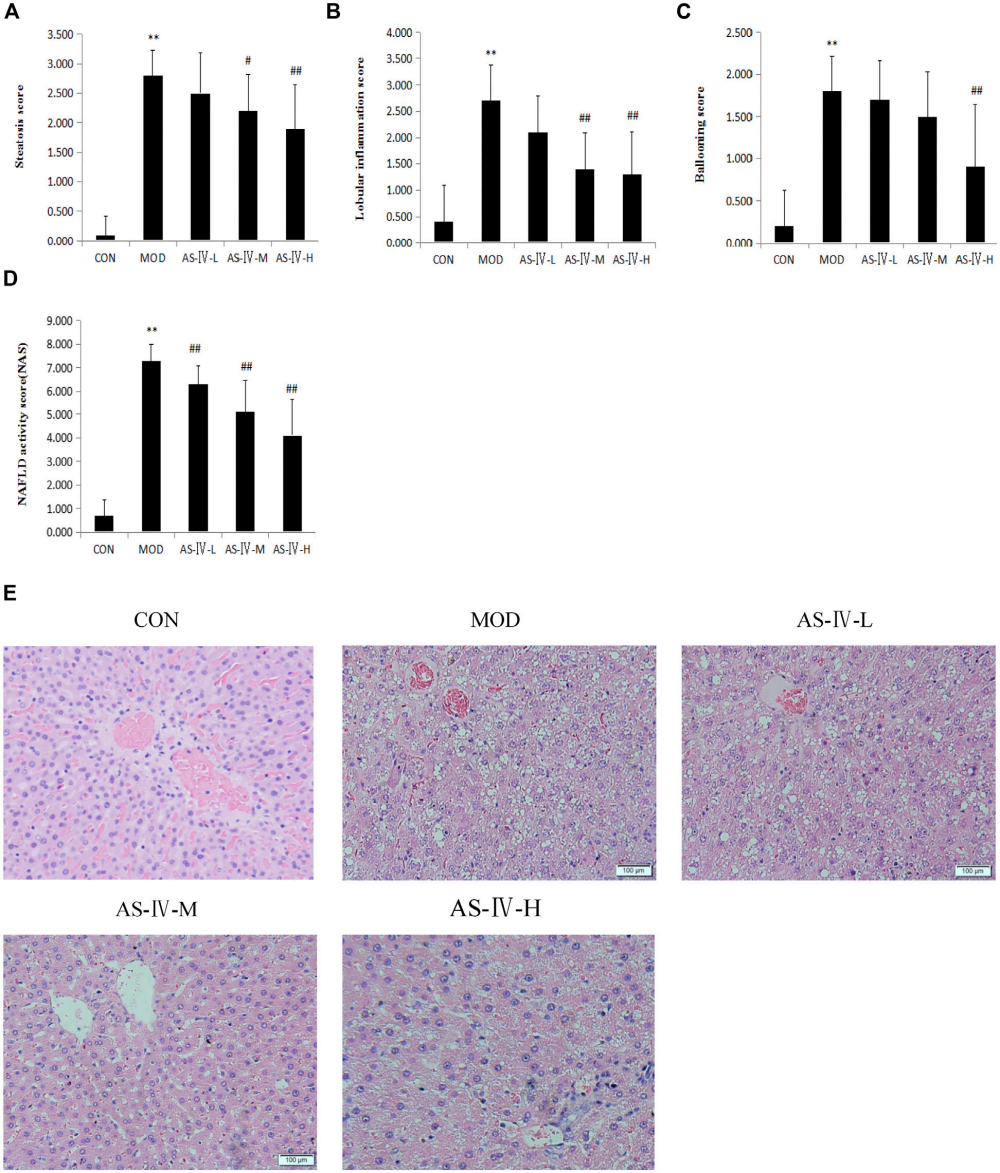
Effects of astragaloside IV on liver histology in HFD-fed rats. **(A)** Steatosis score of rat liver. **(B)** Lobular inflammation score of rat liver. **(C)** Ballooning score of rat liver. **(D)** NAFLD activity score of rat liver. **(E)** Representative histological change of steatosis in liver sections stained with H&E (magnification 400×). Data present the mean±SD. Comparisons between two groups use *t*-tests, and data comparison among multiple groups use one-way ANOVA. *p < 0.05 and **p < 0.01 compared with the control group; ^#^ *p* < 0.05 and ^##^ *p* < 0.01 compared with the model group. CON, control group; MOD, model group; AS-IV-L, astragaloside IV low-dose group; AS-IV-M, astragaloside IV middle-dose group; AS-IV-H, astragaloside IV high- dose group.

NAFLD is characterized by liver fat deposition. Long-term liver fat deposition could lead to liver inflammation. After 12 weeks of HFD, all the sacrificed rats showed liver fat deposition by the liver H&E staining method. This confirmed that the NAFLD rat model was successfully established. After 4 weeks of administration of AS-IV, the liver lipid deposition of NAFLD rats was reduced through microscope observation (Figure 4E). The liver NAS score were markedly decreased in all AS-IV-treated NAFLD rats (Figure 4D). Hepatic steatosis and intralobular inflammation were significantly attenuated in middle- and high-dose AS-IV–treated NAFLD rats (*p* < 0.05) (Figures 4A,B), and balloon-like changes were significantly improved in high-dose AS-IV–treated NAFLD rats (*p* < 0.01) (Figure 4C). The effect of AS-IV on NAS of the liver in NAFLD rats also seemed to be dose-dependent. Through this experiment, we directly confirmed the therapeutic effect of AS-IV on NAFLD rats.

### Astragaloside IV Inhibits Hepatic TLR4, MyD88, and NF-κB Expression in NAFLD Rats

**FIGURE 5:**
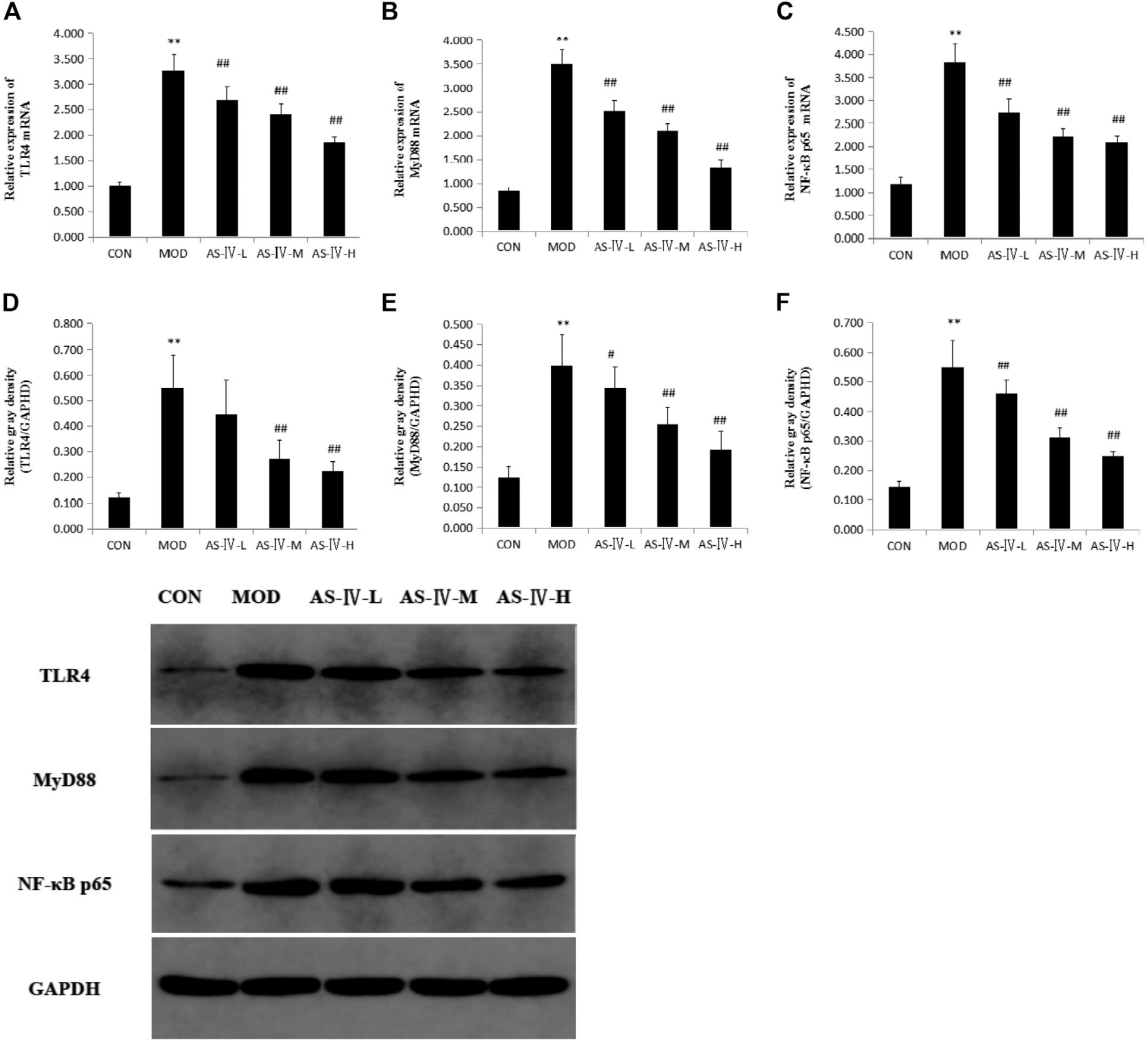
Effects of astragaloside IV on expression of hepatic TLR4, MyD88, and NF-κB p65 in NAFLD rats. **(A)** TLR4 mRNA expression. **(B)** MyD88 mRNA expression. **(C)** NF-κB p65 mRNA expression. **(D)** TLR4 protein expression. **(E)** MyD88 protein expression. **(F)** NF-κB p65 protein expression. **(G)** Representative protein expression bands of hepatic TLR4, MyD88, and NF-κB p65 were analyzed by Western blotting. Data present the mean ± SD. Comparisons between two groups use *t*-tests, and data comparison among multiple groups use one-way ANOVA.*p < 0.05 and **p < 0.01 compared with the control group; ^#^ *p* < 0.05 and ^##^ *p* < 0.01 compared with the model group. CON, control group; MOD, model group; AS-IV-L, astragaloside IV low-dose group; AS-IV-M, astragaloside IV middle-dose group; AS-IV-H, astragaloside IV high-dose group.

Previous discussions have shown that AS-IV may act on the TLR4 signaling pathway in some diseases and TLR4 plays an important role in the development of NAFLD. To determine whether the AS-IV liver histopathology improving effect was associated with TLR4 signaling pathway, we examined the TLR4 mRNA, MyD88 mRNA, and NF-κB mRNA levels of the liver in NAFLD rats. We found the levels of TLR4 mRNA, MyD88 mRNA, and NF-κB mRNA in the liver tissue of NAFLD rats were markedly upregulated compared to normal diet–fed rats (*p* < 0.01). This confirmed that TLR4 signaling pathway plays a role in the pathogenesis of NAFLD. After treated with AS-IV for 4 weeks, the levels of TLR4 mRNA, MyD88 mRNA, and NF-κB mRNA in the liver tissue of NAFLD rats were markedly restored at low dose, middle dose, and high dose of AS-IV. Compared to normal diet–fed rats, the Western blot showed that the protein expression of TLR4, MyD88, and NF-κB in the liver of NAFLD rats was markedly upregulated and restored after AS-IV administration at middle dose and high dose (Figure 5).

### Astragaloside IV Reduces Serum TNF-α, IL-6, and IL-8 Levels in NAFLD Rats

**FIGURE 6:**
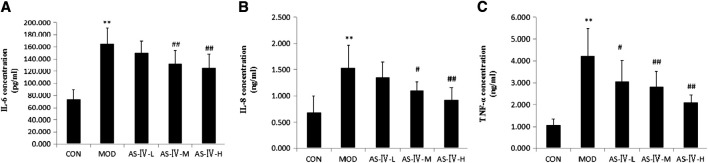
Effects of astragaloside IV on inflammatory factors in NAFLD rats. **(A)** Serum IL-6 concentration. **(B)** Serum IL-8 concentration. **(C)** Serum TNF-α concentration. Data present the mean ±SD. Comparisons between two groups use *t*-tests, and data comparison among multiple groups use one-way ANOVA. *p < 0.05 and **p < 0.01 compared with the control group; ^#^ *p* < 0.05 and ^##^ *p* < 0.01 compared with the model group. CON, control group; MOD, model group; AS-IV-L, astragaloside IV low-dose group; AS-IV-M, astragaloside IV middle-dose group; AS-IV-H, astragaloside IV high-dose group.

As we described in the previous introduction, TLR4 can initiate a series of injury-related immune responses (Erridge, 2010; Wakefield et al., 2010). The overexpression of TLR4 signaling pathway will induce immune inflammatory response. To detect the immune inflammatory response in NAFLD rats, we examined the serum levels of TNF-α, IL-6, and IL-8.We found NAFLD rats showed significantly higher serum levels of TNF-α, IL-6, and IL-8 than the control group. Treatment with different doses of AS-IV significantly reduced the serum TNF-α levels of NAFLD rats (*p* < 0.05), and this effect seemed to be dose-dependent. The serum levels of IL-6 and IL-8 in NAFLD rats were significantly decreased in the middle- and high-dose AS-IV–treated groups (*p* < 0.05) (Figure 6).

## Discussion

Present studies have shown that TLR4 was associated with hepatic steatosis and NAFLD (Miura and Ohnishi, 2014). Singh et al. (2011) found that TLR4 mRNA and protein levels in the liver tissue of NASH patients were higher than those in normal population, and the functionally impaired TLR4 mutant mice were resistant to diet-induced NAFLD (Csak et al., 2011). Enterogenic endotoxin LPS, the ligand of TLR4, increased significantly in NAFLD rodent models induced by different diets (Miura and Ohnishi, 2014). Furthermore, injection of LPS in NAFLD mice could increase proinflammatory cytokines and aggravate the hepatic steatosis (Kudo et al., 2009). Recently, Feng et al. (2019) reported that NAFLD models had been established by feeding high-fat diet to ApoE^−/−^ mice, with impaired intestinal mucosal barrier, increased serum LPS levels. They also found that expressions of TNF-α mRNA, IL-1β mRNA, TLR4, MyD88, and NF-κB protein of the liver tissue were upregulated. In our study, we found that TLR4 mRNA, MyD88 mRNA, NF-κB mRNA, and their proteins were significantly upregulated in the NAFLD rat model rats induced by high-fat diet. These suggested that TLR4/NF-κB signaling pathway was involved in the pathogenesis of NAFLD.

TLR4/NF-κB signal transduction pathway is one of the important pathways associated with inflammatory response. Its activation can lead to a large number of expressions of inflammatory cytokines and then induce a series of inflammatory responses. Cytokines are related closely to NASH. Multiple cytokines, including TNF-α, IL-6, and IL-8, involve in the development of NASH (Mendez-Sanchez et al., 2020). In all of them, TNF-α is the first proinflammatory cytokine released in the body's immune response, which further recruits varieties of inflammatory factors and initiates the development of NAFLD (Stojsavljevic et al., 2014). TNF-α has a strong inhibitory effect on lipoprotein lipase, which can reduce the decomposition of peripheral adipose tissue, promote the synthesis of TG in hepatocytes, and induce lipid accumulation in the liver (Giby and Ajith, 2014; Mendez-Sanchez et al., 2020). TNF-α can hinder insulin signaling by inducing the expression of signal transduction inhibitor 3, leading to insulin resistance (IR) (Neuschwander-Tetri, 2010; Tilg and Moschen, 2010). IL-6 is mainly secreted by adipose tissue and highly expressed in plasma and liver tissues of NASH patients (Schleicher et al., 2015). Studies have shown that IL-6 could impede insulin receptor signaling, lead to IR, and aggravate NAFLD development by inhibiting the expression of IRS-1, GLUT4, and phosphatidylinositol 3-kinase (Park et al., 2010; Wided et al., 2014). In NASH patients, IL-8 levels were significantly elevated, which could induce intrahepatic neutrophil infiltration and lead to hepatocyte injury through neutrophil activation and chemotaxis. (Joshi-Barve et al., 2007; Nassir and Ibdah, 2014). Furthermore, IL-8 can activate liver macrophages and promote liver fibrosis/cirrhosis in NASH patients (Zimmermann et al., 2011). In this study, we found that TLR4 mRNA, MyD88 mRNA, NF-κB mRNA, and their proteins were significantly upregulated in the NAFLD rat model rats induced by high-fat diet. Also, serum TNF-α, IL-6, and IL-8 levels were significantly increased in these rats. These suggested that TLR4/NF-κB signaling pathway and its downstream inflammatory cytokines were involved in the pathogenesis of NAFLD.

Present studies revealed that AS-IV had multiple biological activities, such as anti-inflammatory, antioxidation, lipid-regulating, hypoglycemic, and immunomodulating activities (Ren et al., 2013; Li et al., 2017). Recent studies had shown that AS-IV could protect multiple organs by regulating TLR4/NF-κB signaling pathway. Yang et al. (2013) reported that AS-IV could prevent isoproterenol-induced myocardial hypertrophy in rats by inhibiting the expression of TLR4/NF-κB signaling pathway and its downstream inflammatory cytokines. Lu et al. (2015) reported that AS-IV could downregulate TLR4/NF-κB signaling pathways and inhibit apoptosis, thus alleviatie myocardial injury in the rat model of myocardial ischemia/reperfusion. Leng et al. (2018) reported that AS-IV could improve hyperglycemia-induced vascular endothelial dysfunction and reduce IL-6 and TNF-α levels by regulating TLR4/NF-κB signaling pathways.

However, the effects of AS-IV on NAFLD have been rarely reported. Jiang et al. (2008) reported that AS-IV could alleviate IR and improve liver steatosis in the rat model of type 2 diabetes mellitus. Meanwhile, AS-IV could reduce free fatty acid–induced lipid accumulation in rat hepatocytes. Wu et al. (2016) reported that AS-IV improve lipid metabolism in obese mice by attenuating leptin resistance and regulating the mice heat production network. Zhou et al. (2017a) reported that AS-IV attenuated free fatty acid–induced endoplasmic reticulum stress and lipid accumulation in hepatocytes through adenosine 5′-monophosphate–activated protein kinase (AMPK) activation. Recently, Wang et al. (2018) reported that AS-IV could inhibit IR and lipid accumulation in HepG 2 cells by activating AMPK and reducing phosphorylation of sterol-regulatory element binding proteins ((SREBP)-1c). All of these suggested that AS-IV was a promising drug for the treatment of NAFLD.

In our study, we confirmed that AS-IV administration could reduce dyslipidemia and improve hepatic steatosis in HFD-induced NAFLD rats. These suggested that AS-IV could improve HFD-induced hepatic steatosis and NAFLD. Furthermore, we found that AS-IV could downregulate the expressions of TLR4 mRNA, MyD 88 mRNA, NF-κB p65 mRNA, and their proteins in the liver tissue of HFD-induced NAFLD rats, and, meanwhile, reduce serum TNF-α, IL-6, and IL-8 levels. These suggested that AS-IV may have protective function on NAFLD rats by downregulating TLR4, MyD 88, and NF-κB expression and inhibiting TNF-α, IL-6, and IL-8 levels.

However, this study did not clearly define the detailed mechanisms of how AS-IV inhibits TLR4/NF-κB signaling pathway activation. Our findings are also restricted to animal study and should be confirmed in an vitro study. As mentioned above, the bioavailability of AS-IV oral administration is very low and most of it is excreted by feces. In the gastrointestinal tract, AS-IV will interact with intestinal flora, so we supposed that AS-IV may play a role in the treatment of NAFLD by regulating the structure and function of intestinal microflora and then decrease the levels of enterogenic endotoxin LPS. Therefore, further studies should be conducted in vivo and in vitro to elucidate the beneficial effects of AS-IV on hepatic steatosis.

## Conclusion

In summary, we demonstrated in this study that AS-IV was an effective treatment on HFD-induced NAFLD rats by improving hepatic steatosis and hepatic lipid deposition. This work revealed that AS-IV could inhibit serum TNF-α, IL-6, and IL-8 levels and downregulate the expressions of TLR4 mRNA, MyD88 mRNA, NF-κB mRNA, and their proteins. AS-IV may be a potential drug for the treatment of NAFLD by regulating TLR4/NF-κB signaling pathways.

## Data Availability

The original contributions presented in the study are included within the article/Supplementary Material, and further inquiries can be directed to the corresponding author.
