# Supplementary figures and images for: Astragaloside IV Improves High-Fat Diet–Induced Hepatic Steatosis in Nonalcoholic Fatty Liver Disease Rats by Regulating Inflammatory Factors Level via TLR4/NF-κB Signaling Pathway
Source: Front Pharmacol. 2021 Jan 29;11:605064. doi: 10.3389/fphar.2020.605064 (PMC7941269; doi:10.3389/fphar.2020.605064)

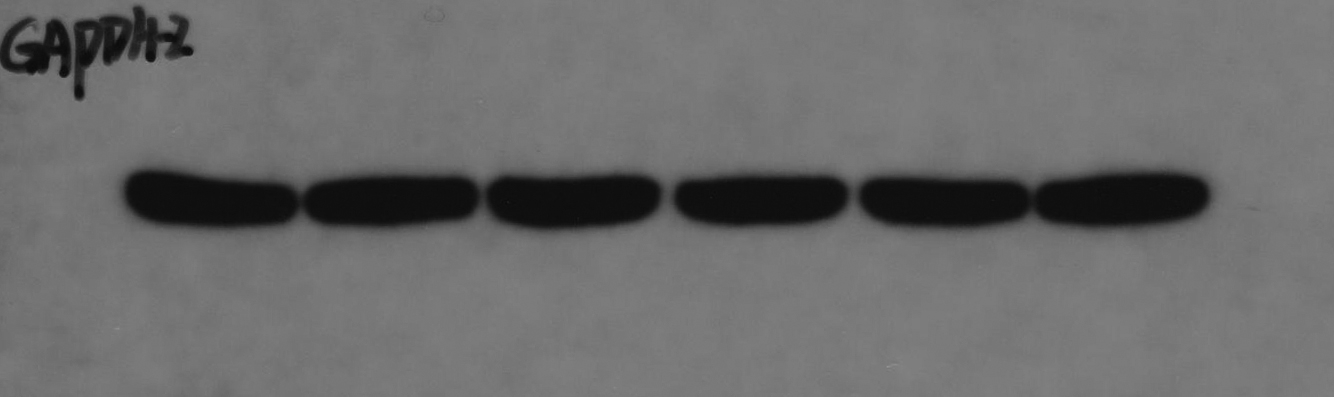

Supplement: Supplementary file 1 [file datasheet1.zip › 605064 Western blot original image/GAPDH.JPG]

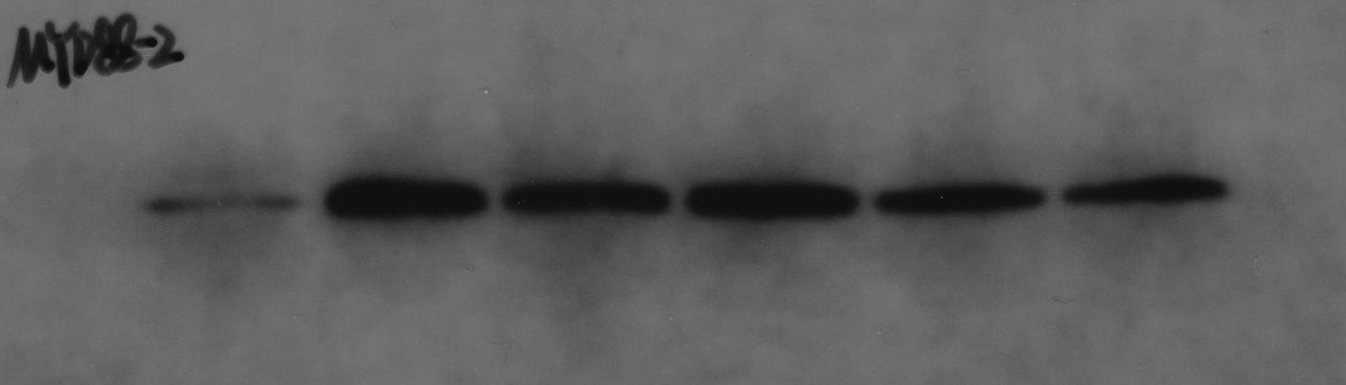

Supplement: Supplementary file 1 [file datasheet1.zip › 605064 Western blot original image/MyD88.JPG]

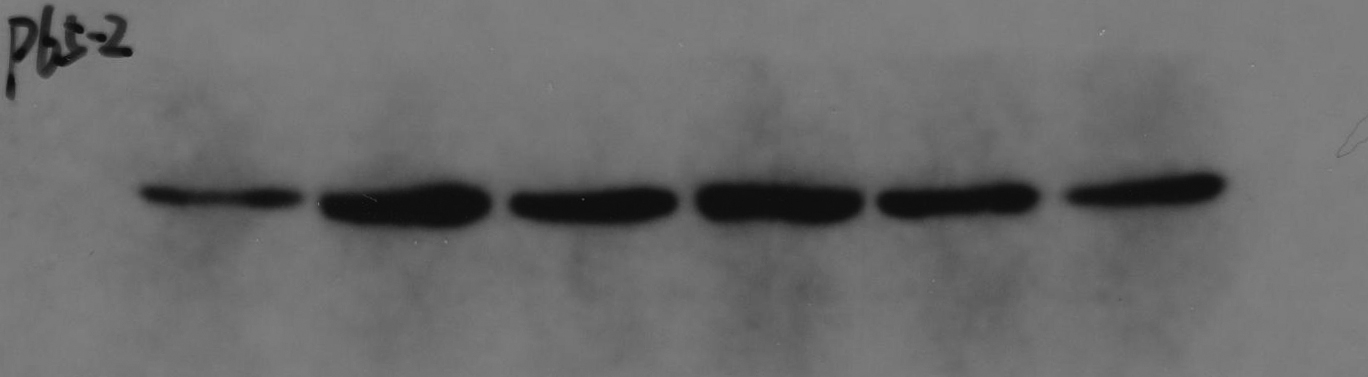

Supplement: Supplementary file 1 [file datasheet1.zip › 605064 Western blot original image/NF-kBp65.JPG]

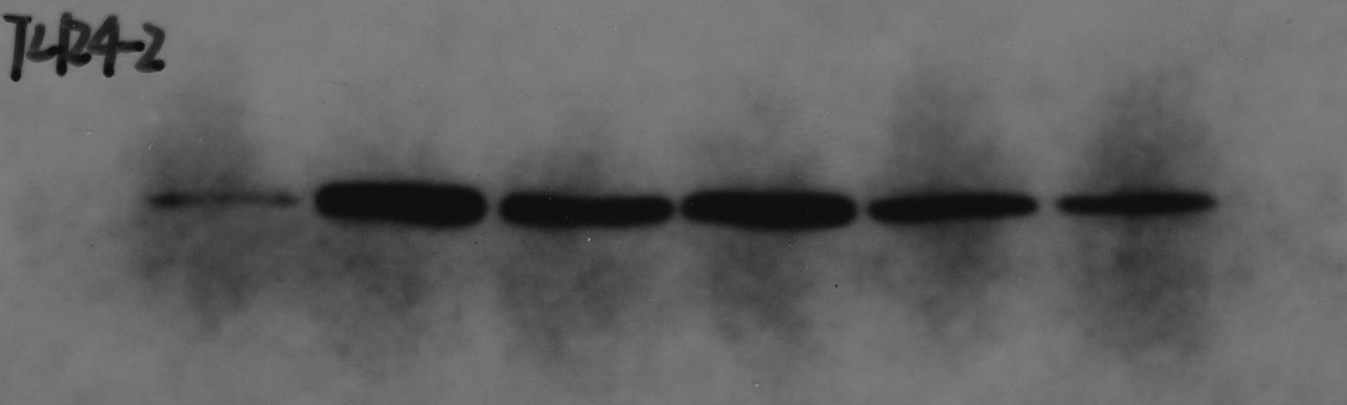

Supplement: Supplementary file 1 [file datasheet1.zip › 605064 Western blot original image/TLR4.JPG]
